# Blistered Skin

**Published:** 1867-12

**Authors:** 


BLISTERED SKIN, taking out the inflammation, cooling the parts and restoring them to their natural condition.

